# EHMTI-0111. Intra-variability of migraine attacks features

**DOI:** 10.1186/1129-2377-15-S1-D70

**Published:** 2014-09-18

**Authors:** M Viana, G Sances, N Ghiotto, E Guaschino, M Allena, M Allena, G Nappi, C Tassorelli, PJ Goadsby

**Affiliations:** 1 Headache Science Center, C. Mondino National Neurological Institute, Pavia, Italy; 2 Headache Group – NIHR-Wellcome Trust Clinical Research Facility, King’s College London, London, UK

## Background

Migraine attacks may have different feature with respect to different patients but also within the same patient. At the best of our knowledge in literature is not described the percentage of patients that report attacks as stereotyped and the percentage of patients reporting attacks with different phenotype.

## Aim

To evaluate the percentage of migraine patients reporting attacks with same characteristics on three consecutive attacks.

## Methods

Each patient recorded in a diary the features of three consecutive attacks. Characteristics recorded were: pain intensity on a 4 point scale, presence of nausea (N), vomiting (V), photophobia (PT), phonophophia (PN), osmophobia (O), allodynia (A), cranial autonomic symptoms (CAS) (at least one), premonitory symptoms (at least one). Patients were allowed to take a medication (triptan).

## Results

In 30 patients, nobody presented identical characteristic on the three studied attacks. Results remained the same if we do not consider the pain intensity and the presence of at least one premonitory symptoms (0 out of 30 patients). If we consider only the presence of associated symptoms (N, V, PT, PN, O, A, CAS), 3 out of 30 patients had the same phenotype on three different attacks, while if we consider only the presence/absence of N, V, PT, PN, 9 out of 30 patients (30%) had three identical attacks. Triptan intake occurred at a mean of 63 minutes after pain onset when the average pain intensity was 2.

## Conclusion

Migraine attacks are very different not just among patients, but also in the same patient. Our data indicates that stereotypy of attacks is uncommon.

No conflict of interest.

